# A novel entomological index, *Aedes aegypti* Breeding Percentage, reveals the geographical spread of the dengue vector in Singapore and serves as a spatial risk indicator for dengue

**DOI:** 10.1186/s13071-018-3281-y

**Published:** 2019-01-08

**Authors:** Janet Ong, Xu Liu, Jayanthi Rajarethinam, Grace Yap, Derek Ho, Lee Ching Ng

**Affiliations:** 1 Environmental Health Institute, National Environment Agency, Singapore, Singapore; 2 Environmental Public Health Operations, National Environment Agency, Singapore, Singapore; 3 School of Biological Sciences, Nanyang Technological University, Singapore, Singapore

**Keywords:** *Aedes aegypti*, Dengue, Entomological index, Spatial risk indicator, Geographical expansion

## Abstract

**Background:**

*Aedes aegypti* is an efficient primary vector of dengue, and has a heterogeneous distribution in Singapore. *Aedes albopictus*, a poor vector of dengue, is native and ubiquitous on the island. Though dengue risk follows the dispersal of *Ae. aegypti*, the spatial distribution of the vector is often poorly characterized. Here, based on the ubiquitous presence of *Ae. albopictus*, we developed a novel entomological index, *Ae. aegypti* Breeding Percentage (BP), to demonstrate the expansion of *Ae. aegypti* into new territories that redefined the dengue burden map in Singapore. We also determined the thresholds of BP that render the specific area higher risk of dengue transmission.

**Methods:**

We performed analysis of dengue fever incidence and *Aedes* mosquito breeding in Singapore by utilizing island-wide dengue cases and vector surveillance data from 2003 to 2013. The percentage of *Ae. aegypti* breeding among the total *Aedes* breeding habitats (BP), and the reported number of dengue fever cases in each year were calculated for each residential grid.

**Results:**

The BP of grids, for every year over the 11-year study period, had a consistent positive correlation with the annual case counts. Our findings suggest that the geographical expansion of *Ae. aegypti* to previously “non-dengue” areas have contributed substantially to the recent dengue fever incidence in Singapore. Our analysis further indicated that non-endemic areas in Singapore are likely to be at risk of dengue fever outbreaks beyond an *Ae. aegypti* BP of 20%.

**Conclusions:**

Our analyses indicate areas with increasing *Ae. aegypti* BP are likely to become more vulnerable to dengue outbreaks. We propose the usage of *Ae. aegypti* BP as a factor for spatial risk stratification of dengue fever in endemic countries. The *Ae. aegypti* BP could be recommended as an indicator for decision making in vector control efforts, and also be used to monitor the geographical expansion of *Ae. aegypti*.

**Electronic supplementary material:**

The online version of this article (10.1186/s13071-018-3281-y) contains supplementary material, which is available to authorized users.

## Background

Dengue fever has caught global attention due to its increasing frequency of major epidemics in recent years. It is estimated that 2.5 billion individuals, residing in more than 100 countries, are at risk of dengue fever, with an annual case burden of approximately 50–100 million infections [1]. Facilitated by the rapid urbanization and increased global travel, dengue fever continues to make its geographical spread across the world.

Dengue fever is caused by the dengue virus (DENV), exists as four different serotypes, DENV1-4. Infection with one serotype confers lifelong immunity only to that particular serotype [2]. Hence, repeated outbreaks in a given population at short intervals are generally due to different serotypes. All four dengue serotypes can be found co-circulating in Singapore at all times [3]. It has been observed previously that there is a cyclical pattern of outbreaks that peaks every five to seven years in Singapore [4, 5].

Dengue fever is transmitted to humans by *Aedes* spp. mosquitoes, mainly by *Ae. aegypti*. *Aedes albopictus* has also been incriminated as an important vector of DENV. In Hawaii, parts of China, Seychelles, and more recently, France, where *Ae. aegypti* was absent or had limited presence, *Ae. albopictus* was identified as the main vector in the dengue outbreaks [6, 7]. However, reported transmission due to *Ae. albopictus* is typically less intense and short-lived [8].

In Singapore, the vector status of *Ae. aegypti* and *Ae. albopictus* reflects the global situation. Dengue transmission, as indicated by a cluster of cases located within a 150 m radius and with onset of illness within a 14-day period, co-locates with presence of *Ae. aegypti* [9, 10]. There is no evidence of transmission in places with only *Ae. albopictus*. The vector status of the two *Aedes* mosquitoes is corroborated by vector surveillance studies using Gravitraps, which found that mosquitoes caught in areas with clusters of dengue cases were predominantly *Ae. aegypti* [11]. Potentially infective *Aedes*, as demonstrated by the presence of dengue viruses in the head of the mosquito, were found to be *Ae. aegypti* [12]. *Aedes aegypti*, found in urbanized built-up areas, is thus the primary vector of DENV in the country. *Aedes aegypti* is known to originate from Africa and was introduced into the coastal cities of South East Asia around 19th century *via* the shipping industry [13]. The precise time of its arrival to Singapore is not known. In contrast, *Ae. albopictus* is native and ubiquitous throughout Singapore, due in part to the abundant greenery that lines the streets and adorns the housing estates.

Despite a low *Aedes* house index of around 2%, Singapore continues to experience regular outbreaks [14]. In 2005, 2007 and 2013, Singapore experienced explosive dengue fever outbreaks that resulted in 14,032, 8287 and 22,170 indigenous cases, respectively, with incidence rates of 322.5, 180.6, and 404.9 per 100,000 population [15–17]. All three outbreaks were associated with the replacement of predominant DENV serotypes, which was believed to have played an important role in the escalating number of cases [15, 18].

Factors that may have contributed to the population’s sensitivity to outbreaks include: (i) rapid increase in population, which grew from 2.1 million in 1970 to 5.4 million by 2013; (ii) rapid urbanization with an extensive transport network of 164 km of expressway and 199.6 km of mass rapid transit (MRT) lines across the 720 km^2^ island; (iii) increased globalization as demonstrated by 26.5 million air arrivals in 2013, as compared to 1.7 million recorded in 1970; (iv) low herd immunity, especially among younger generations, due to decades of low local transmission; and (v) presence of cryptic breeding sites which are difficult to identify and henceforth implement vector control [19–27]. Some of these developments have undoubtedly favoured the expansion of *Aedes* population as well as the frequent importation of new viruses and rapid dispersal of viruses within the country, all of which facilitate human, vector and virus encounters [28–30]. Expansion of *Ae. aegypti* to historically ‘non-dengue’ areas in the country has the potential to expose an immunologically naïve human population to DENV, increasing the risk of outbreaks.

Vector surveillance, recommended by the World Health Organization (WHO), is a routine practice in many dengue endemic countries to provide quantifiable measure of fluctuations in magnitude of dengue vector populations [31, 32]. Globally, the most commonly used indices for vector surveillance are House Index (HI), Container Index (CI) and Breteau index (BI). In Singapore, HI has been used for monitoring the *Ae. aegypti* population in the community. However, Singapore’s vector control programme has brought the HI down from about 50% in the 1960s to 0.30% in the 2000s, way below the target levels for HI set by the WHO [9, 11, 33]. The low *Aedes* HI has rendered HI insensitive for gauging *Ae. aegypti* population in the community and hence, HI is no longer sensitive for dengue risk assessment. This study therefore aims to introduce a novel vector index based on routine inspection data, with higher spatial resolution and better relevance to spatial dengue transmission risk.

## Methods

### Data collection and preparation

Georeferenced data on dengue fever cases and *Aedes* spp. larval counts from routine surveillance in the main island of Singapore from 2003 to 2013 were extracted from the Geographical Information System (GIS) Database of the National Environment Agency (NEA), Singapore. The daily-updated database is part of the national vector control programme. Dengue is a notifiable disease in Singapore. It is mandatory for medical practitioners and clinical laboratories to notify all clinically diagnoses and laboratory-confirmed dengue cases to the Ministry of Health (MOH), Singapore [34]. The laboratory confirmation of clinical diagnoses is achieved through either NS1 antigen detection or viral RNA detection by PCR or IgM detection [35, 36]. The notification information included demographic data, travel history, onset of illness, residential and workplace address. Dengue cases were tagged to the address, either residential or workplace address, after epidemiological investigation had been carried out by officers to determine and confirm the location where the cases acquired dengue. The *Aedes* larval surveillance data are derived from daily inspection of residential and non-residential premises for mosquito breeding in habitats such as drains, gutters and containers, conducted by approximately 1000 vector control officers. These inspections include those scheduled for regular preventive surveillance, and those conducted in response to dengue transmission in a location. If mosquito breeding was discovered or suspected, a georeferenced sample would be taken and sent to NEA laboratory for species identification. The *Aedes* larvae were identified by trained taxonomists using morphological keys [37]. A breeding site was defined as a positive *Ae. aegypti* breeding site if the sample was identified to contain at least one *Ae. aegypti* larva. We also obtained data of annual total population, resident population and residential dwelling units from reports published by the Singapore Department of Statistics [20, 38].

As administrative areas of Singapore are of irregular shapes and varying sizes, we superimposed a 1 × 1 km grid system on the map of the main island and used these fixed grid cells as study units to minimize the normalization required. We choose 1 × 1 km to balance the need for a relatively large area of each study unit and a large sample size at the same time, given Singapore’s size. Grids were categorized into non-residential and residential based on the land use in 2013 as determined by where the centroids of grids fall within. After excluding major industrial and forested areas, 213 residential grids were included in the analyses.

### Definition and estimation of *Ae. aegypti* Breeding Percentage (BP)

*Aedes aegypti* Breeding Percentage (BP) was defined as the proportion of *Ae. aegypti* positive breeding sites of the total number of *Aedes* spp. positive breeding sites found (*Ae. aegypti* and *Ae. albopictus*) in a defined area within a defined time period:

$$ \mathrm{BP}=\left(\mathrm{No}.\mathrm{of}\  Ae. aegypti\ \mathrm{positive}\ \mathrm{breeding}\ \mathrm{sites}/\mathrm{No}.\mathrm{of}\  Ae des\ \mathrm{spp}.\mathrm{positive}\ \mathrm{breeding}\ \mathrm{sites}\right)\times 100 $$

BP, expressed as a percentage, assumes the ubiquitous presence of native *Ae. albopictus* population in Singapore, and uses total *Aedes* breeding for the normalization of field data in order to cancel out the sampling error from non-systematic inspection and cryptic breeding sites. Only positive breeding sites were taken to avoid inclusion of grids with no data which could arise because the grids have not been visited or checked thoroughly by the field inspectors. This seeks to address non-systematic operational inspection, which tends to bias towards outbreak areas.

To estimate the yearly BP for each grid, we mapped the location of *Aedes* breeding sites onto each grid and extracted the number of *Ae. aegypti* and/or *Ae. albopictus* breeding sites found within each grid, for each year from 2003 to 2013. Due to the fact that breeding data are the result of non-probability sampling, BP estimated by percentage of *Ae. aegypti* breeding among all *Aedes* breeding found could incur a large error, especially when the denominator was small. Here, we set the threshold of denominator to be ten, after examining the distribution of the number of *Aedes* breeding sites found. BP for grids with at least ten *Aedes* breeding found in a year was calculated by definition. For other grids, the small number of breeding sites gives low confidence, BP were thus estimated using ordinary Kriging with a spherical variogram model (Additional file 1: Figure S1). BP in residential grids were compared graphically year to year to assess the change in geographical distribution of *Ae. aegypti* population over the years. Temporal trend of BP was assessed by the median BP of each year.

### Estimation and analysis of case burden

To estimate yearly dengue case burden for each grid, dengue fever cases from 2003 to 2013 were plotted spatially onto the grids. Case burden in residential grids was plotted as pixel images and compared yearly to assess the change in geographical distribution of dengue transmission over the years. Temporal trend of case burden was assessed by median case count of each year. Some analyses involving case burden used the transformed variant of case count to stabilize the variance across different scales. The transformation used was f (Y) = log (Y + 1) where Y is the grid case count.

### Association between BP and case burden

The spatial relationship between BP and case count was assessed by Spearman’s correlation between grid BP and grid case count. The correlation was analyzed separately for each year to check for consistency and robustness of the relationship.

### Determination of BP thresholds for risk stratification

To use BP in novel dengue areas as a risk flag for preventive surveillance, we defined case burden as a categorical variable. For a particular year, a grid was classified as high dengue burden if it contributed at least 0.5% of all cases, moderate dengue burden if it contributed 0.1–0.5%, and low dengue burden if less than 0.1%. For example, if a grid reported less than 22 cases in 2013, it was labelled as low burden area in 2013. Therefore, each grid scored 11 class labels of “high burden”, “moderate burden” or “low burden” during the 11-year study period. The accuracy of using BP to discriminate these areas was determined based on the standard values of area under the ROC curve (AUC): AUC < 0.5 (not discriminative); 0.5 < AUC < 0.7 (less discriminative); 0.7 < AUC < 0.9 (moderately discriminative); and 0.9 < AUC < 1.0 (highly discriminative).

### Alignment between increase in BP and increase human population and number of housing units

The alignment between increase in BP and increase in human population and residential dwelling units was assessed by Spearman’s correlation, in attempt to provide an explanation for the increased BP.

### Data analysis

Data and statistical analyses were conducted using R version 3.1.1 [39]. R packages utilized in the study include *sp*, *gstat*, *sciplot* and *pROC* [40–43]. All methods used for analyses are described in respective sections. *P-*values of Spearman’s correlation tests were computed *via* the asymptotic t-approximation. A significance level of 0.05, and with Bonferroni correction in the case of multiple comparisons, was utilized in this study.

## Results

In the present study, we used a novel entomological index, *Ae. aegypti* BP, which has consistent positive spatial correlation with localized dengue fever burden to demonstrate the geographical expansion of *Ae. aegypti* in Singapore. This simple index uses field entomological data that need not be consistently and systematically collected. Analysis using the BP further revealed that the geographical expansion of *Ae. aegypti* has contributed to the recent increase and altered corresponding distribution of dengue cases in Singapore.

### Association between BP and case burden

**Fig. 1:**
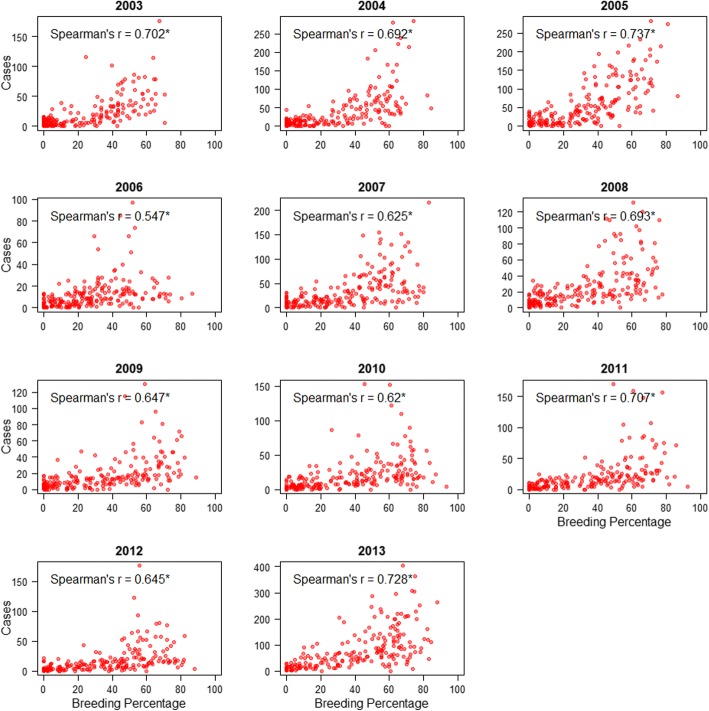
Relationship between Breeding Percentage (BP) and case count assessed by Spearman**’**s rank correlation test. Asterisks indicate statistical significance

Every geographical grid was tagged with case count and BP for each of the 11 years. BP was consistently positively associated with case count, though the Spearman’s correlation coefficient varies across the years, ranging between 0.547–0.737 (Fig. 1). All correlations were significant (*P* < 0.0001) with respect to the significance level of 0.05.

### Determination of BP thresholds for risk stratification for dengue control

**Fig. 2:**
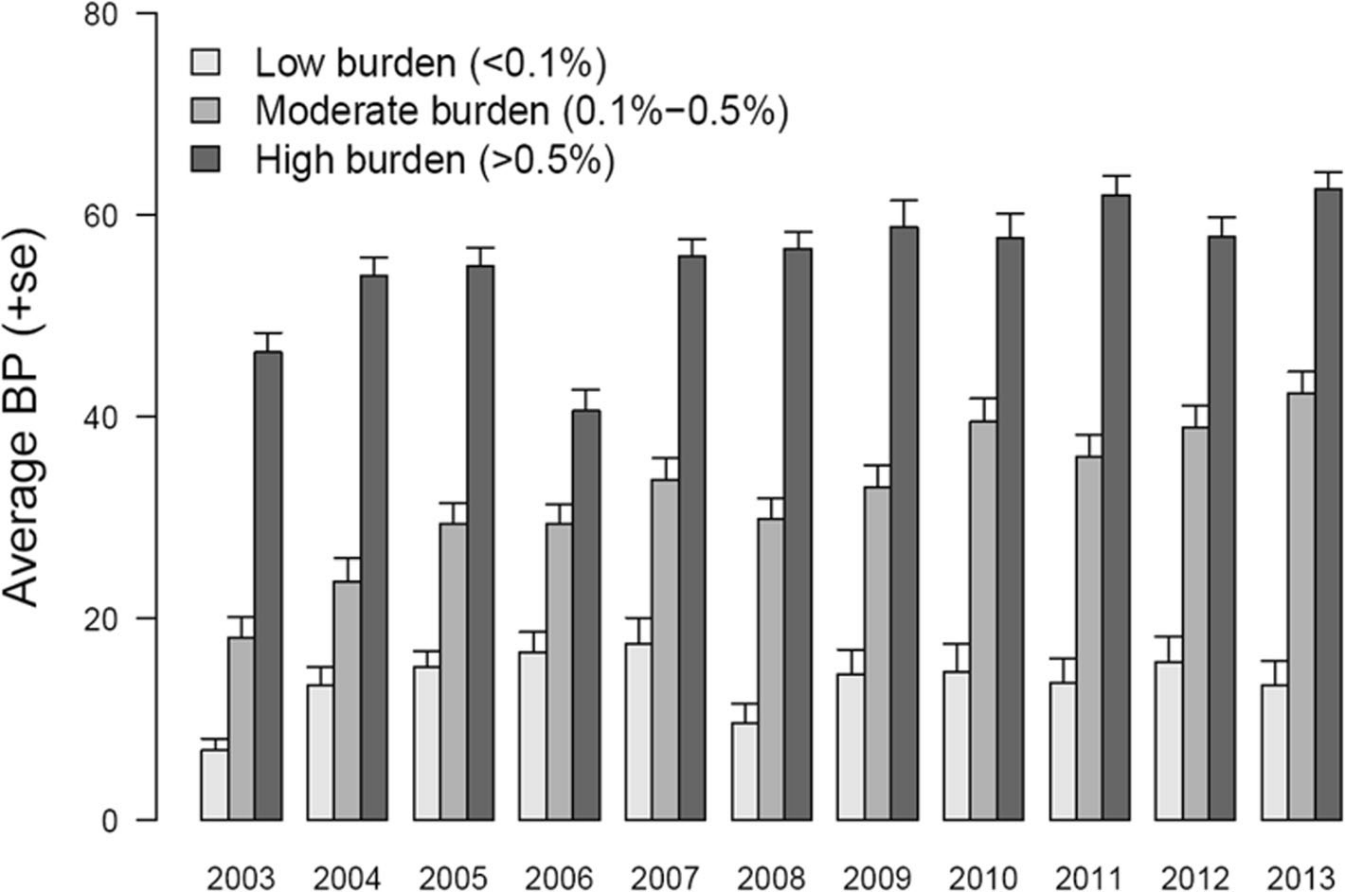
Differences in Breeding Percentage (BP) between low burden, moderate burden and high burden areas. Average BP for low burden (light grey bars), moderate burden (grey bars) and high burden (dark grey bars) areas are shown with standard error bars

Based on the contribution to national burden of dengue, the grids were stratified into three levels of disease burden and their average BP were calculated yearly. Through the 11 years, each of the high burden grids (> 0.5% of national annual cases) was characterized by BP of range 40–60%, moderate burden grids (0.1–0.5% of national annual cases) were associated with BP of 20–40%, mostly more than 30%, and low burden grids (< 0.1% of national annual cases) had BP less than 20% (Fig. 2). As a result, grids with BP less than 20% were considered “low risk”, BP of 20–40% as “moderate risk”, and BP above 40% as “high risk”.

**Fig. 3:**
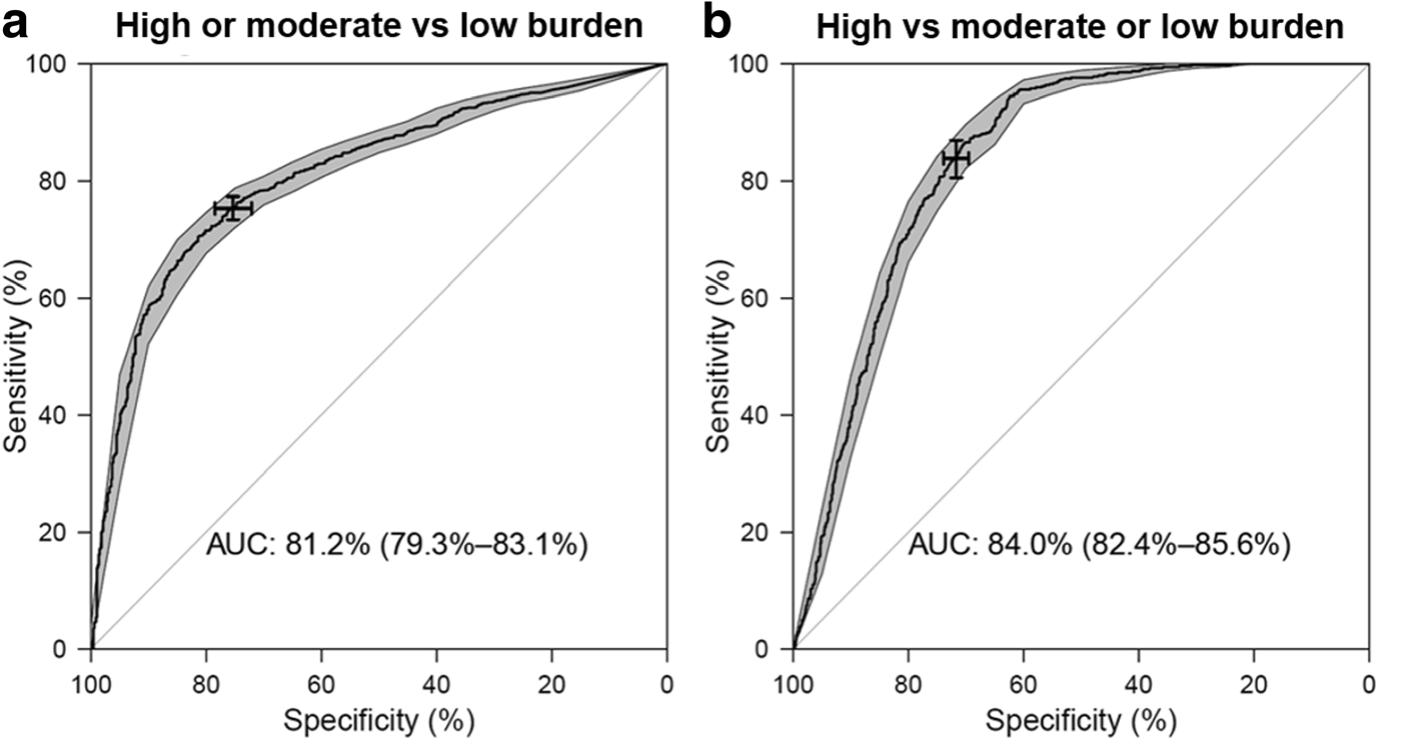
Receiver operating characteristics curves (black thick line) of classification of areas as “high-burden”, “moderate burden” and “low burden” by Breeding Percentage, and the 95% CI (grey shaded area) of sensitivity over a selected set of specificity. **a** Classification of “high-burden” or “moderate burden” *versus* “low burden”. The area under the curve is 0.812 with 95% CI: 0.793–0.831. The sensitivity-specificity combination given by the chosen threshold 20% is indicated on the curve: sensitivity = 75.28% (95% CI: 73.22–77.22%); specificity = 75.31% (95% CI: 71.89–78.57%). **b** Classification of “high-burden” *versus* “moderate burden” or “low burden”. The area under the curve is 0.840 with 95% CI: 0.824–0.856. The sensitivity-specificity combination given by the chosen threshold 40% is indicated on the curve: sensitivity = 83.85% (95% CI: 80.55–86.97%); specificity = 71.69% (95% CI: 69.52–73.86%)

Figure 3 shows the ROC curve of using BP to categorize risk of dengue transmission. For classification of low dengue burden *versus* other categories, the AUC was 0.812 (95% CI: 0.793–0.831), indicating moderately discriminative capability of BP. Using a low BP as threshold to predict dengue risk was less sensitive and highly specific, whereas using high BP, though highly sensitive, resulted in low specificity. Setting the 20% BP as a threshold to predict dengue risk resulted in 75.31% specificity and 75.28% sensitivity. The 20% BP was based on the cut-off between the low and moderate burden grids. At higher level of 40% derived from BP of high burden grids, specificity was 71.69% and sensitivity was 83.85%.

### Spatial and temporal characteristics of BP

**Fig. 4:**
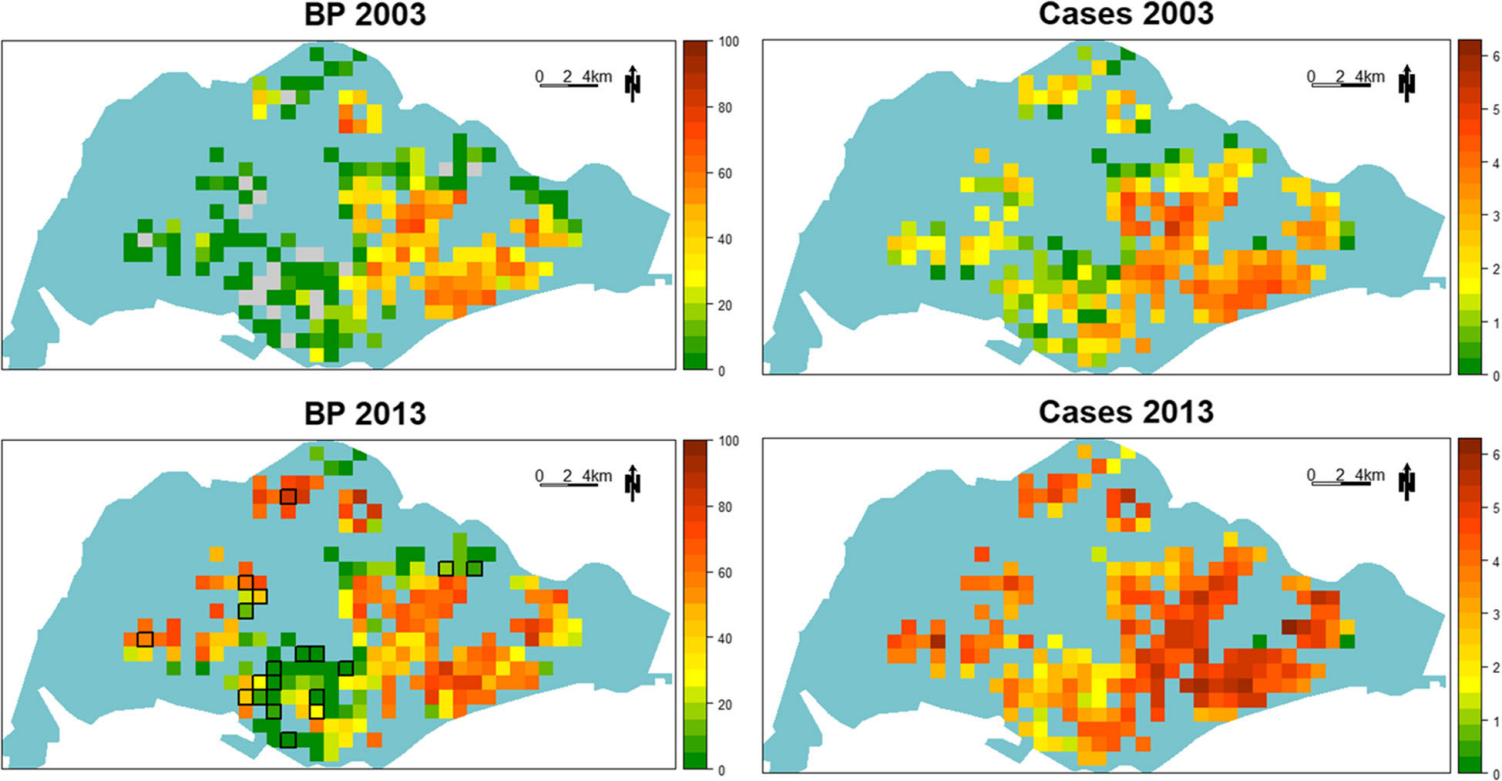
Spatial distribution of Breeding Percentage (BP) and transformed dengue case burden in 2003 and 2013. Left panels: values of BP are color-coded, with difference shades of green indicating BPs < 20%, yellow shades indicating BPs between 20% and 40%, and orange through dark red indicating BPs ≥ 40%. Residential grids with BP 0% in 2003 were highlighted in gray and outlined in black in the 2013 map. In 2003, areas with higher BP were exclusively in the eastern part of the island. By 2013, *Ae. aegypti* has expanded into the northern and western part of the island. The spatial expansion of *Ae. aegypti* is also illustrated by areas that turn from a BP of 0% to having a BP of > 0%. Furthermore, areas that had relatively higher BPs in 2003 also registered higher levels of BPs in 2013, as visualized by the color gradient. Right panels: transformed dengue case burden in residential grids in 2003 and 2013 are represented by colors, with green being the least dengue burden and dark red being the highest burden. Vertical comparisons illustrate the spatial expansion of *Ae. aegypti* and dengue transmission. Horizontal comparisons reveal a likely association between BP and dengue transmission

Graphical presentation of BP in 2003 and 2013 (Fig. 4, left panels) showed that in 2003, *Ae. aegypti* was present in moderate and high proportion (BP ≥ 20%), mostly in the central and eastern part of Singapore. However, by 2013, grids with BP ≥ 20% were found in the western part and north corner. Areas in the central and eastern part also showed higher BP values in 2013. The right panels of Fig. 4 show the corresponding spread of dengue from the central and eastern part of Singapore to the northern and western parts. Of significance are areas that had evolved from low risk to high risk in the last 11 years. These areas include the Jurong and Choa Chu Kang areas around 2004–2005; Clementi, Bukit Batok and Pasir Ris areas around 2006 and 2007; Queenstown and Central areas during 2008–2012; and the Upper Thomson area around 2013.

**Fig. 5:**
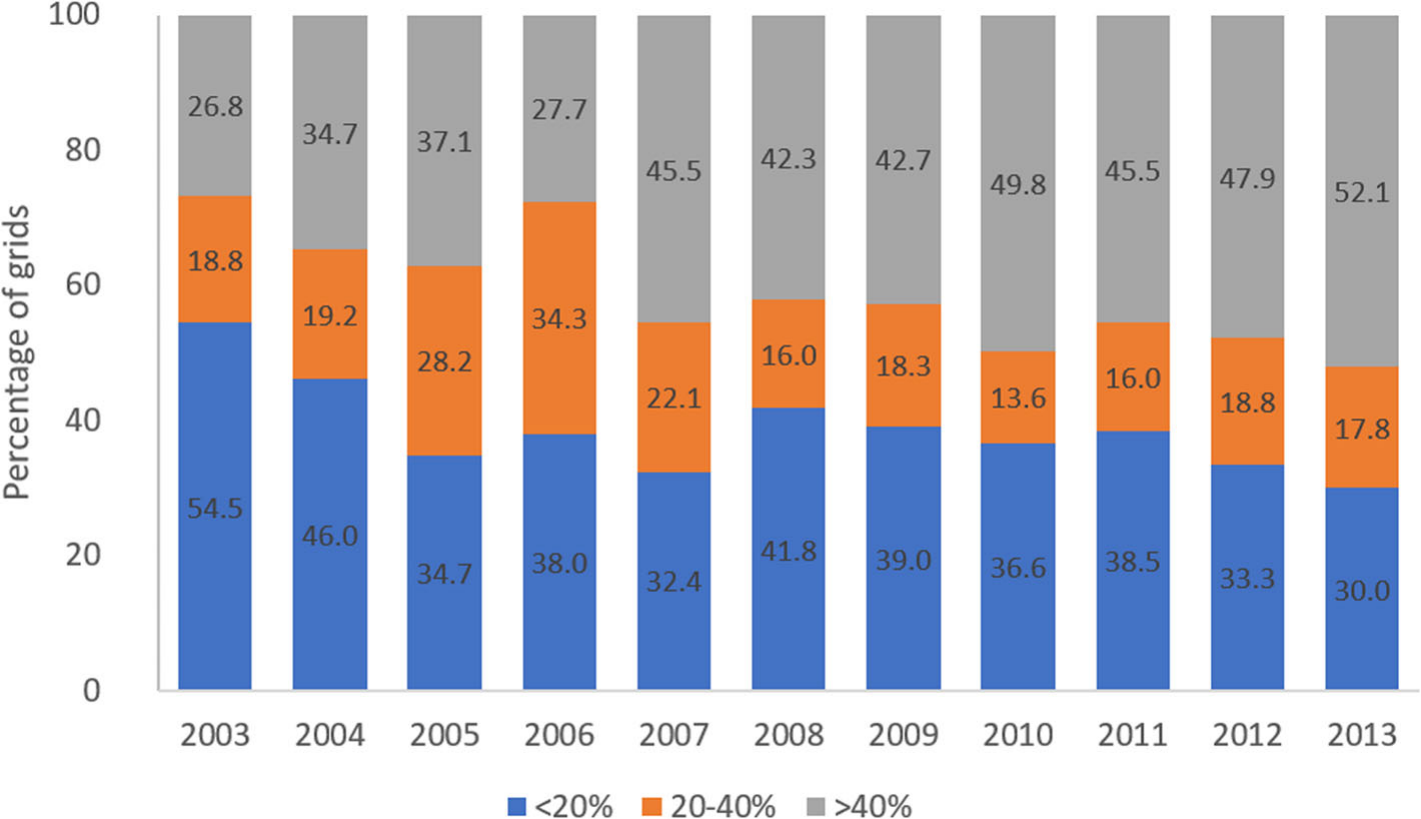
Grids with different range of Breeding Percentage (BP)

**Fig. 6:**
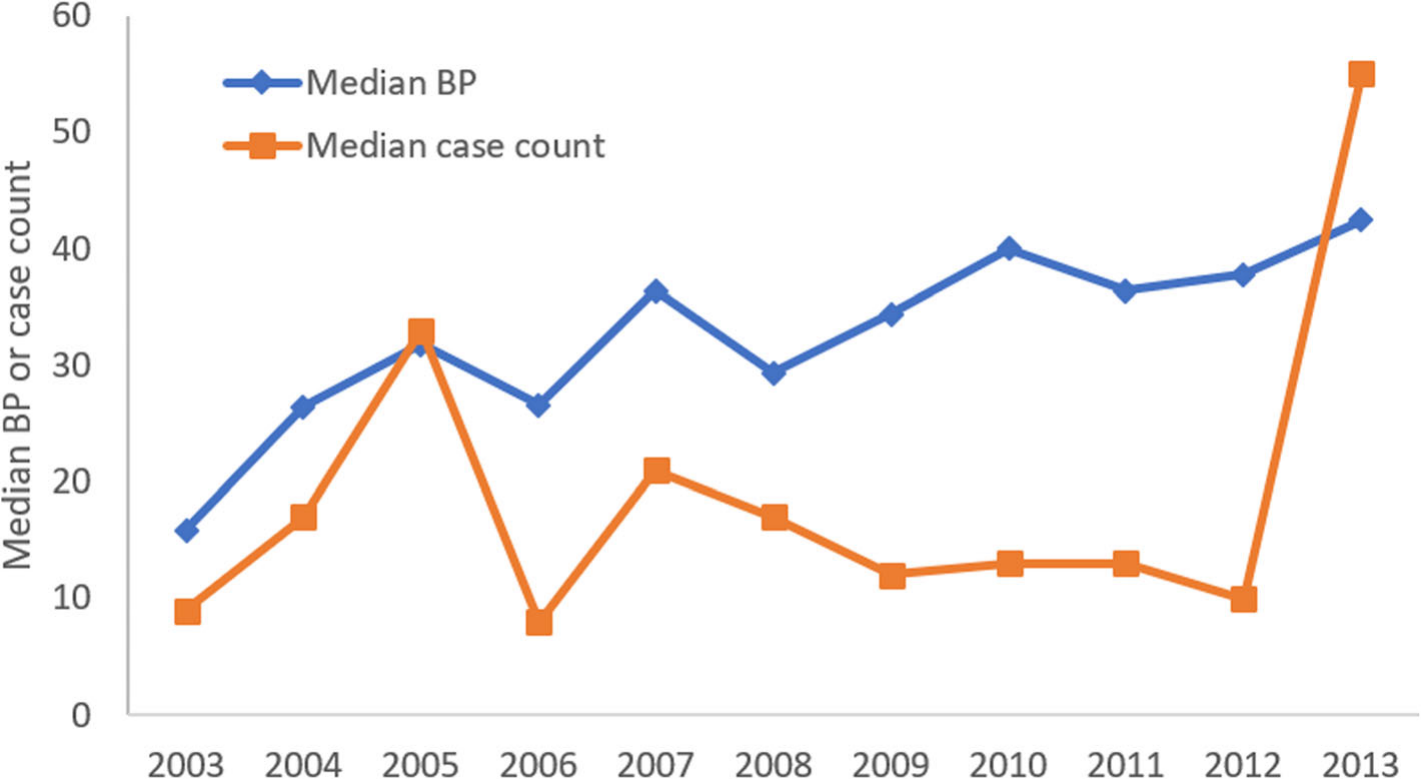
Annual median grid Breeding Percentage (BP) and median grid case count. Median BP increased over time, regardless of lull (2008–2012) or outbreak years

In general, there has been an increase in the percentage of geographical grids that show moderate or high level of BP (Fig. 5). In 19 of the grids, *Ae. aegypti* was not found in 2003 (BP = 0%) but was found to have BP ranging between 5–80% by 2013. This demonstrates the spatial expansion of *Ae. aegypti* over the 11-year period. At the same time, the median grid BP increased from 15.87% in 2003 to 42.47% in 2013 (Fig. 6), indicating a general increasing trend of BP over time, regardless of lull or outbreak (2005, 2007 and 2013) years.

### Alignment between increase in BP and increase in human population and number of housing units

**Fig. 7:**
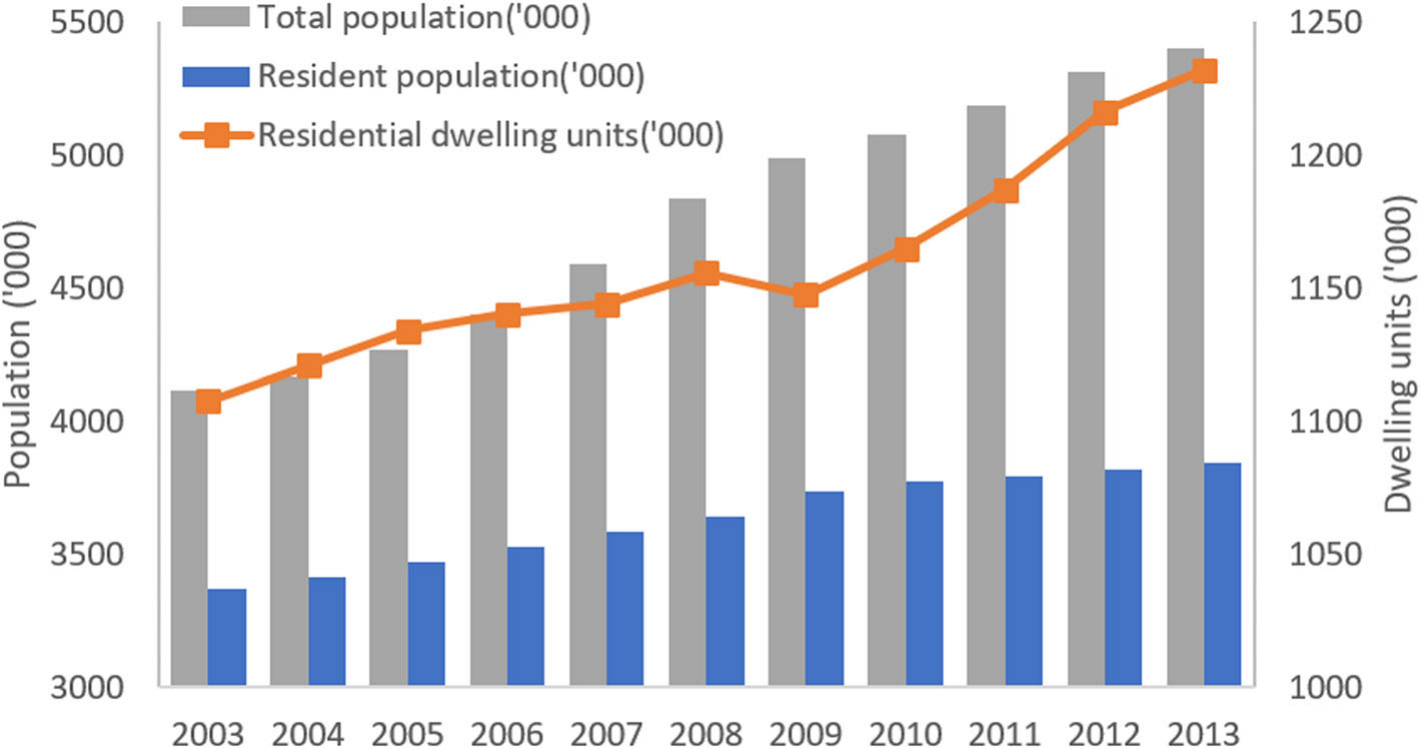
Population and residential dwelling units in Singapore from 2003 to 2013

**Fig. 8:**
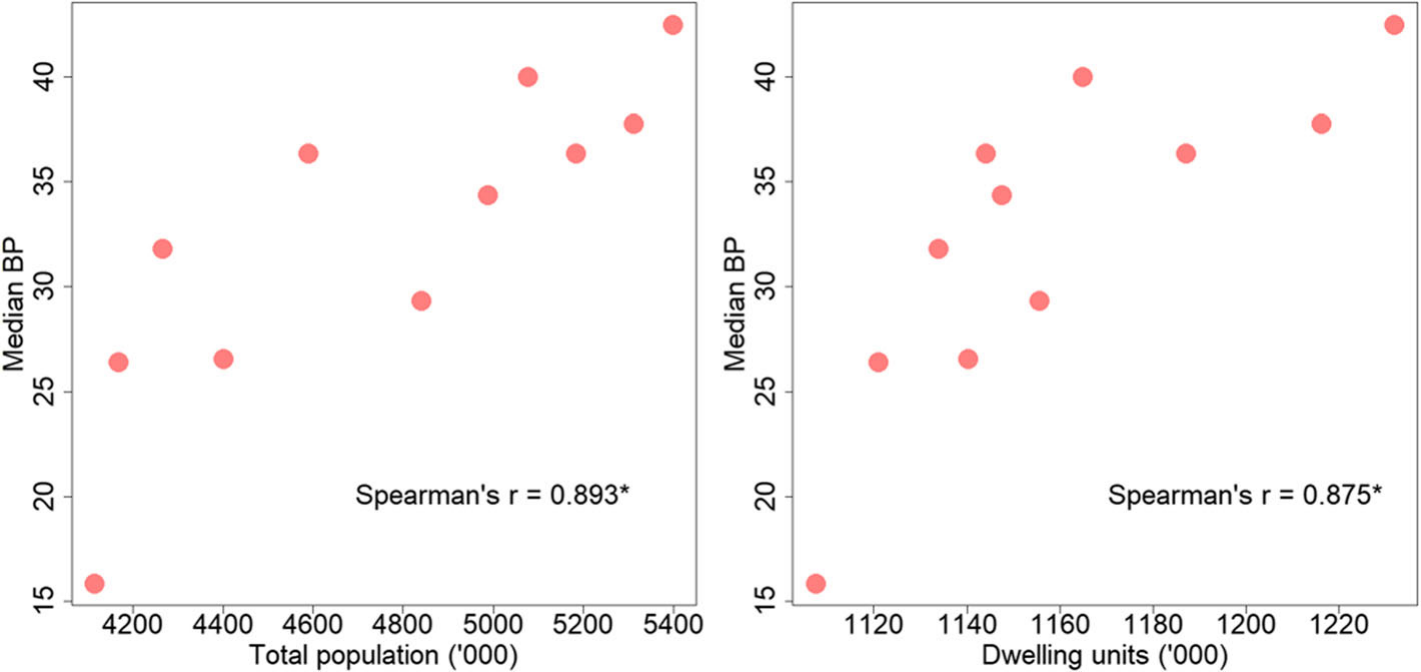
Relationship between Median Breeding Percentage (BP) with total population and number of residential dwelling units, assessed by Spearman**’**s correlation. Asterisks indicate statistical significance

Increase in BP corresponds to increase in human population and number of residential dwelling units (Fig. 7). The annual median BP has a strong significant positive correlation with total population (Spearman’s correlation: *r*_(11)_ = 0.893, *P* < 0.0001) and the number of residential dwelling units (Spearman’s correlation: *r*_(11)_ = 0.875, *P* < 0.0001) (Fig. 8).

## Discussion

Vector indices are key risk factors in dengue transmission. Commonly used indices are house index, container index and Breteau index [1]. Globally these indices have been used for risk assessment and early warning for dengue epidemics [44, 45]. However, there is little solid evidence to quantify the relationship between these indices and dengue transmission [46]. In Singapore, the House Index has been used by authorities for dengue risk assessment since 1960s and it has been brought down and maintained at low levels by NEA’s intensive vector control programme [14]. In spite of the consistent low HI, Singapore remains susceptible to dengue outbreaks, suggesting that the low HI is no longer sensitive for risk assessment. It highlights the need for a new vector index that accounts for the spatial heterogeneity of dengue transmission risk.

This study introduced a new entomological index, the *Ae. aegypti* Breeding Percentage (BP), presented its association with case burden, and proposed thresholds for its applications as a risk indicator for dengue control interventions. Absolute number of reported cases was used to measure case burden instead of incidence rate because control interventions are directly related to case count.

*Aedes aegypti* BP, reported in the present study was developed using the existing *Aedes* breeding data obtained through the routine vector surveillance programme in Singapore. As routine larval collection efforts are not uniform spatially as well as temporally, using the absolute data for risk assessment would be biased. The development of *Ae. aegypti* BP takes into consideration the ubiquitous presence of *Ae. albopictus* and normalizes the data with the total *Aedes* breeding sites, which comprises of *Ae. aegypti* and *Ae. albopictus* breeding sites. When compared with the traditional *Aedes* HI, which is reported only on a national level, the main strength of BP is its higher resolution and usefulness for spatial analyses of dengue transmission. We have shown that on a yearly basis, areas with higher BP tend to have higher case count based on historical data. Therefore, BP can be used as a risk factor in spatial risk mapping of dengue transmission together with other factors.

The new index has also demonstrated the expansion of *Ae. aegypti* into new territories from 2003 to 2013. The strong correlation between annual median BP and annual total population and residential dwelling units suggests the expansion might be a result of rapid population growth and urbanization. However, more thorough analysis is needed to pinpoint the causes conclusively. These new territories have become highly vulnerable to dengue fever outbreaks, probably contributed by the minimal prior exposure of inhabitants to DENV. The low level of immunity to DENV has been demonstrated by our seroprevalence studies [47].

Cases in areas where *Ae. aegypti* was absent occurred as isolated cases throughout the years, with no evidence of temporal and spatial link among these cases. This could be due to cases acquiring dengue outside of home and workplace (where cases were tagged to), which is not unexpected considering the dynamic human movement in a city. Alternatively, these small number of cases could be due to the low transmission of *Ae. albopictus*, which is recognised as a secondary vector of dengue globally.

Thresholds were derived with respect to Singapore’s dengue situation and vector population, to ease the application of BP in guiding decisions on the ground to allocate vector control resources. In areas where *Ae. aegypti* is entrenched (BP ≥ 20%), more resources should be assigned for intensive vector control. In areas where *Ae. aegypti* has recently infiltrated (BP < 20%), the focus could aim at elimination of the vector. In more recent years, BP at the national level has been incorporated into a dengue forecast model, which has been useful in predicting dengue trends at least 3 months ahead [48]. BP of each grid has also been incorporated into a spatial risk map which is developed annually, to guide prioritization of resources [49]. These are part of a general effort to stratify risk of dengue temporally and spatially [50].

In view of how chikungunya has adapted to *Ae. albopictus*, and the increasing number of reports of *Ae. albopictus*-driven dengue outbreaks internationally, the epidemiology could be dynamic and demands close monitoring to ensure that any change in vector status is detected [51, 52].

## Conclusions

With the disease burden of dengue fever increasing across the world, a similar approach would be applicable to other dengue endemic areas, where *Ae. albopictus* is prevalent, e.g. most of tropical and sub-tropical Asia. The *Ae. aegypti* BP could be recommended as an indicator for decision making in vector control efforts. It can also be used to monitor the geographical expansion of *Ae. aegypti*.

## Additional file

Additional file 1: **Figure S1.** Empirical variogram (circle) and modelled spherical variogram (line) for the residuals of the Breeding Percentage. (TIF 245 kb)

## Abbreviations

BI: Breteau index
BP: Breeding percentage
CI: Container index
DENV: Dengue virus
GIS: Geographical information system
HI: House index
MOH: Ministry of Health
NEA: National Environment Agency
WHO: World Health Organization
