# Supplementary figures and images for: A novel entomological index, Aedes aegypti Breeding Percentage, reveals the geographical spread of the dengue vector in Singapore and serves as a spatial risk indicator for dengue
Source: Parasit Vectors. 2019 Jan 8;12:17. doi: 10.1186/s13071-018-3281-y (PMC6325748; doi:10.1186/s13071-018-3281-y)

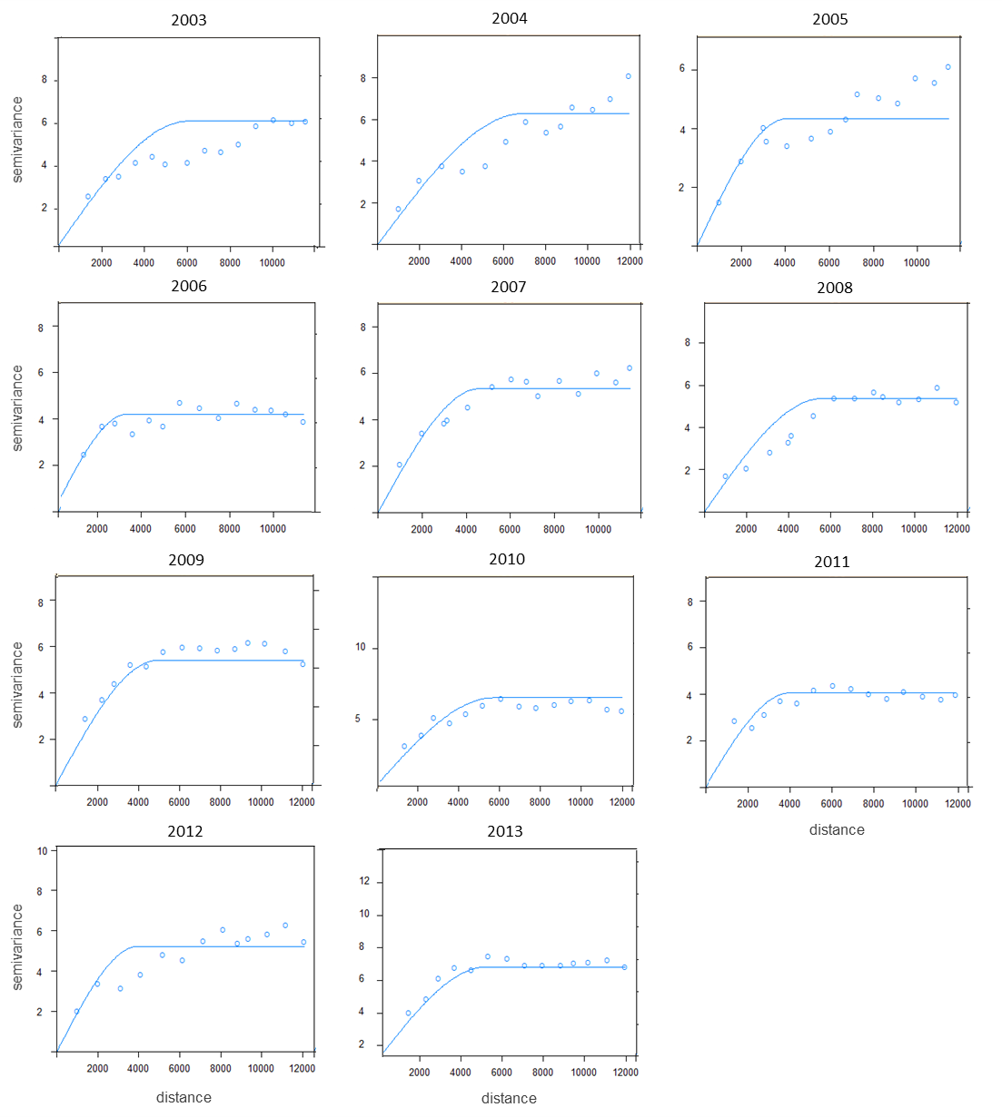

Supplement: Supplementary file 1 — Figure S1. Empirical variogram (circle) and modelled spherical variogram (line) for the residuals of the Breeding Percentage. (TIF 245 kb) [file 13071_2018_3281_MOESM1_ESM.tif]
